# Simulation methods to estimate design power: an overview for applied research

**DOI:** 10.1186/1471-2288-11-94

**Published:** 2011-06-20

**Authors:** Benjamin F Arnold, Daniel R Hogan, John M Colford, Alan E Hubbard

**Affiliations:** 1 Division of Epidemiology, School of Public Health, University of California, Berkeley, CA, USA; 2 Center for Health Decision Science, Harvard School of Public Health, Boston, MA, USA; 3 Division of Biostatistics, School of Public Health, University of California, Berkeley, CA, USA

**Keywords:** Computer Simulation, Power, Research Design, Sample Size

## Abstract

**Background:**

Estimating the required sample size and statistical power for a study is an integral part of study design. For standard designs, power equations provide an efficient solution to the problem, but they are unavailable for many complex study designs that arise in practice. For such complex study designs, computer simulation is a useful alternative for estimating study power. Although this approach is well known among statisticians, in our experience many epidemiologists and social scientists are unfamiliar with the technique. This article aims to address this knowledge gap.

**Methods:**

We review an approach to estimate study power for individual- or cluster-randomized designs using computer simulation. This flexible approach arises naturally from the model used to derive conventional power equations, but extends those methods to accommodate arbitrarily complex designs. The method is universally applicable to a broad range of designs and outcomes, and we present the material in a way that is approachable for quantitative, applied researchers. We illustrate the method using two examples (one simple, one complex) based on sanitation and nutritional interventions to improve child growth.

**Results:**

We first show how simulation reproduces conventional power estimates for simple randomized designs over a broad range of sample scenarios to familiarize the reader with the approach. We then demonstrate how to extend the simulation approach to more complex designs. Finally, we discuss extensions to the examples in the article, and provide computer code to efficiently run the example simulations in both R and Stata.

**Conclusions:**

Simulation methods offer a flexible option to estimate statistical power for standard and non-traditional study designs and parameters of interest. The approach we have described is universally applicable for evaluating study designs used in epidemiologic and social science research.

## Background

Estimating the sample size and statistical power for a study is an integral part of study design and has profound consequences for the cost and statistical precision of a study. There exist analytic (closed-form) power equations for simple designs such as parallel randomized trials with treatment assigned at the individual level or cluster (group) level [1]. Statisticians have also derived equations to estimate power for more complex designs, such as designs with two levels of correlation [2] or designs with two levels of correlation, multiple treatments and attrition [3]. The advantage of using an equation to estimate power for study designs is that the approach is fast and easy to implement using existing software. For this reason, power equations are used to inform most study designs. However, in our applied research we have routinely encountered study designs that do not conform to conventional power equations (e.g. multiple treatment interventions, where one treatment is deployed at the group level and a second at the individual level). In these situations, simulation techniques offer a flexible alternative that is easy to implement in modern statistical software.

Here, we provide an overview of a general method to estimate study power for randomized trials based on a simulation technique that arises naturally from the underlying data model typically assumed by power and sample size equations. The method is universally applicable to a broad range of outcomes and designs, and it easily accommodates complex design features such as different follow-up plans, multiple treatment interventions, or different site-specific cluster effects. Simulation can also estimate the expected impact of deviations from optimal study implementation, such as item non-response and participant drop out. Statisticians have estimated design power using computer simulation for decades to benchmark analytic sample size equations [4,5], but most published articles on estimating power using simulation have been either highly specific in application or highly technical [6-15]. Feiveson [16] presents an applied, general overview of estimating power by simulation using Stata software, but the article is not indexed in major databases, and has only been cited twice in applied research [17,18]. To our knowledge, this is the first published application of this approach that outlines the method using the data generating models that are both the foundation of traditional power calculations and familiar to quantitative epidemiologists. Our goal with this article is to motivate and demonstrate with concrete examples how to use simulation techniques to estimate design power in a way that quantitative, applied epidemiologists can use in practice. We believe this approach has the potential for widespread application because the setting in which we have applied it is similar to that found in many studies.

As a motivating example, we recently considered a study design to test the independent and combined effects of environmental interventions (sanitation, handwashing and water treatment) and nutrient supplementation on child growth, measured by length/height. Growth faltering in the first years of life can have profound, negative consequences on lifelong human capital [19]. Enteric infections can cause growth faltering through acute diarrhea and parasitic insults [20,21]. There is abundant evidence that environmental interventions can reduce enteric infections [22-24] and some evidence that they improve growth [25,26]. Interestingly, even the best nutritional interventions fail to eliminate the majority of linear growth faltering typically observed in low-income country populations [27]. Nutritionists have hypothesized that nutrient supplementation interventions could be enhanced by complementary household environmental interventions that reduce fecal bacteria ingestion during the first years of life and potentially improve gut health [28,29].

**Figure 1:**
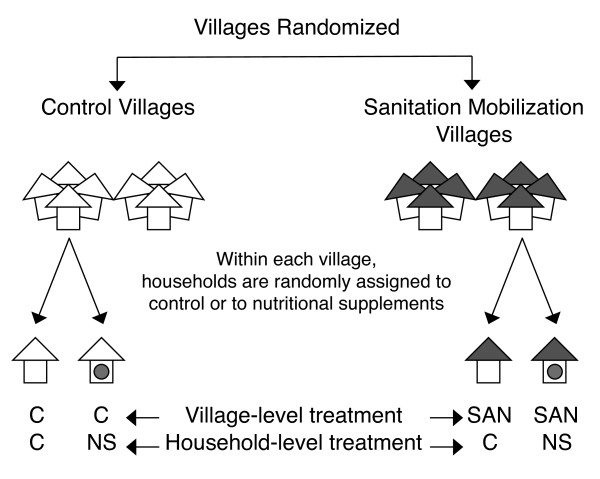
**Schematic of the factorial design described in the introduction**. Village clusters are first randomized to either control (C) or a sanitation marketing treatment (SAN). Then, households with children < 6 months within each village are randomized to control (C) or to daily nutritional supplementation (NS).

To test this hypothesis, we considered a two-treatment factorial trial in rural Bangladesh, where children < 6 months of age are randomly assigned to one of four groups: control (no treatment), sanitation mobilization, lipid-based nutrient supplementation (LNS), and sanitation plus LNS. (The trial is still in the planning stage, and will include additional environmental interventions.) Sanitation mobilization campaigns rely on community-level intervention techniques, such as village-level defecation mapping and 'walks of shame,' under the premise that community-level dynamics help catalyze a shift of community norms away from open defecation and toward using improved sanitation facilities, such as private toilets [30]. LNS is a micro- and macronutrient rich paste that is incorporated into existing meals and is typically administered daily beginning at age 6 months to supplement breast feeding and traditional foods [31-34]. Since sanitation mobilization must be delivered at the community level and spillover effects within a community are almost certain to exist, a cluster-randomized trial would be a natural design of choice [1]. However, due to field logistics, the monetary cost of adding clusters is far higher than the cost of adding households within clusters, and so we considered a design where the sanitation treatment is deployed randomly at the community level, and LNS is deployed randomly to households within each community cluster (Figure 1). Consistent with existing trials of LNS that are randomized at the household level [31-34], we assume no significant spillover effects between households in the same community. Children's length is measured at baseline (pre-treatment) and again after two years of intervention.

This design makes power calculations difficult for two reasons. First, the two treatments are deployed at different levels (community and household) and second, there are two sources of correlation in the outcome: within-community and within-child. Considering the estimating approach that will be used, combined with the complex clustered nature of the data-generating distribution, no analytical solution exists to calculate the statistical power for our hypothesis of interest given this design. Below, we introduce the simulation approach to estimate power, benchmark it against the conventional approach for a simple design, and then return to this example to demonstrate a more complex application. We conclude with a practical discussion of extensions and limitations to the approach, and in supporting files we provide example code to run our simulations in both R and Stata (see additional files 1 and 2: R-programs.pdf, Stata-programs.pdf).

## Methods

The statistical power of design is defined as the complement of the Type II error rate (1-*β*), and is the probability of rejecting the null hypothesis when it is false. Estimating study power requires an investigator to specify a small number of non-design parameters that describe the outcome and expected treatment effect. These parameters may include the mean, variance and expected difference between treatment and control in the outcome variable (the effect size). For cluster randomized trials or trials with repeated measures, an investigator using conventional power equations must also specify the intraclass correlation coefficient (ICC) or its variant, the coefficient of variation, which summarizes the correlation between repeated measures within an experimental unit [1,35]. Power equations for designs with multiple levels of correlation require that investigators specify even more parameters [2,3]. Typically, these parameters are estimated from existing data or extracted from prior published studies. The simulation approach we outline below estimates a related set of parameters and then uses those to simulate outcome data from a specified data-generating model under a null and alternative hypothesis.

As an introductory example, consider a community-randomized intervention trial to evaluate the impact of a sanitation mobilization campaign (as described in the introduction) on child height, where child height is measured once, post-intervention. We measure the outcome, *Y_ij_*, as standardized height-for-age Z-scores (HAZ) for child *j *living in community *i*, and the treatment, *A_i_*, is randomly assigned at the community level with equal probability to half of the enrolled communities. To use simulation, it is necessary to specify the data generating distribution for *Y_ij_*. One such convenient distribution that we use throughout this article is the class of mixed effects models [36], which give rise to conventional power equations for clustered or longitudinal designs [1]. Specifically, we assume that the continuous outcome HAZ (*Y_ij_*) arises from the following model:(1)

There are four parameters in the model: *μ *is the mean HAZ score in the control children, *β*_1 _is the estimated difference in HAZ comparing intervention children (*A *= 1) to the control children (*A *= 0); *b_i _*is a cluster-level random effect and *ε_ij _*is an error term that captures individual-level variability and measurement error. We assume that the random effect and error term are normally distributed with mean zero and known standard deviation, and are uncorrelated: *b_i _*~ N(0, *σ_g _*), *ε_ij _*~ N(0, *σ_e_*), *cov*(*b_i_*, *ε_ij_*) = 0.

The variability of the random effects and residual error relate directly to the ICC because in this model the definition of the ICC is the ratio of between-cluster variability to the total variability [1]:(2)

The model in equation 1, along with the assumptions on the error distribution and the related parameter estimates, are what we use to simulate the outcomes *y_ij_*. (Throughout this article our notation uses capital letters to identify random variables and lower case letters to identify realizations of those random variables: *Y_ij _*is a random variable, and *y_ij _*is a realized outcome drawn from the distribution of *Y_ij_*.) In order to run the simulations, it is necessary to make assumptions about the four parameters (*μ*, *β*_1_, *σ_g_*, *σ_e_*). The effect size (*β*_1_) will likely be specified based on prior studies, subject matter knowledge or the minimum effect size that is either biologically meaningful or cost-effective. We use an existing and representative dataset with HAZ measurements for children nested within clusters (the training dataset) to estimate the remaining three parameters. (When existing data are unavailable, an investigator can also assign values to these parameters from prior studies or subject matter knowledge.) In practice, it is possible to estimate the variability of the cluster and residual random effects with the training dataset by fitting a mixed model of the outcome on an intercept with a random intercept specified for clusters:(3)

where *μ*, *b_i _*and *ε_ij _*are defined above. This is implemented in Stata using the xtmixed command, in SAS with PROC MIXED, and in R with the nlme or lme4 packages (for examples in Stata, see additional file 2: Stata-programs.pdf). The linear mixed model will provide two estimates of variability: cluster-level variability  and residual variability . Importantly, the different levels of variability can only be estimated if the dataset includes repeated observations at each level. For this example, to estimate  repeated measures of *Y_ij _*are required (i.e., HAZ for multiple children within each cluster).

The simulation requires the following steps:

1. Estimate parameters  from a training dataset (described above).

2. Create a population of 2,000 children (2 arms × 100 clusters/arm × 10 children/cluster), with a unique ID variable for each cluster, a unique ID variable for each child and an indicator for assigned treatment: treated (*A *= 1) and control (*A *= 0).

3. Generate a random effect for each cluster (200 total), *b_i_*, which is a draw from a normal distribution with mean 0 and SD .

4. Generate a residual error term for each child, *ε_ij_*, which is a draw from a normal distribution with mean zero and SD .

5. Simulate an outcome for each child, *y_ij_*, using equation 1.

6. Regress *y_ij _*on the treatment indicator *A_i_*, using robust sandwich standard errors [37] to account for clustering at the highest level of correlation, and store the one- or two-sided *P *value for the test .

7. Repeat steps 3 through 6 a large number of times (at least 1,000).

8. The empirical power of the design is the fraction of *P *values that are smaller than 0.05.

**Figure 2:**
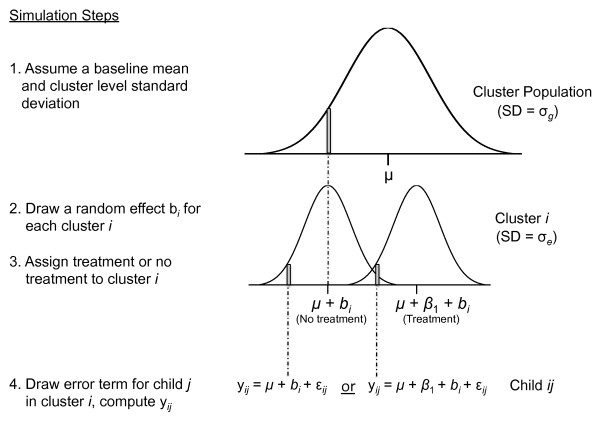
**Summary of random effects draws used in a basic simulation of a cluster-level intervention with individual-level outcomes (*y**_ij_***)**. The population mean is *μ*, *b_i _*are random effects at the cluster level and *ε_ij _*are random errors at the individual level. *β*_1 _is the assumed difference in *y_ij _*for individuals in treated clusters versus individuals in control clusters.

Figure 2 includes a schematic for the simulation process. The fraction of statistically significant *P *values across simulation runs is an estimate of empirical power because simulation runs that fail to identify statistically significant differences are technically Type II errors (since we assume *β*_1 _≠ 0 in the simulation).

Note that in this article's examples we use generalized linear models with robust sandwich standard errors to account for correlation; we could equivalently use a generalized estimating equation (GEE) approach [38] with robust sandwich standard errors. For our specific application, generalized linear models and GEE are useful because they naturally estimate marginal parameters and require investigators to make fewer assumptions about the data generating distribution during parameter estimation than mixed effects models [39]. On a practical level, marginal models are also computationally simpler than mixed models, which is relevant when simulating the analysis thousands of times. However, an advantage of using simulation to estimate power is that investigators can use whatever estimation approach they plan to use in their actual analysis.

It is straightforward to modify the approach for a continuous outcome to accommodate a binomial outcome. For example, in the sanitation mobilization intervention investigators may also want to measure its impact on child diarrhea. Let *p_ij _*be the probability of diarrhea for the *jth *child in the *ith *cluster. Consider a standard logistic model of *p_ij _*:(4)

where *μ *is the log-odds of the baseline probability of diarrhea, *β*_1 _is the log of the odds ratio of diarrhea comparing children in intervention communities (*A *= 1) to the children in control communities (*A *= 0); *b_i _*is a cluster-level random effect. As before, we assume that the random effect is normally distributed with mean zero and known standard deviation: *b_i _*~ N(0, *σ_g_*).

The model in equation 4 is used to simulate a binary outcome that is distributed as a Bernoulli random variable with probability *p_ij_*, and so three parameters must be specified (*μ*, *β_1_*, *σ_g_*). Analogous to the approach for continuous outcomes, we use a training dataset with repeated outcome measurements at the cluster-level to estimate the log-odds of baseline diarrhea prevalence (*μ*), and the cluster-level variability, . It is possible to estimate the variability of the cluster-level random effect by fitting a binomial mixed model of the outcome on an intercept with a random intercept specified for clusters:(5)

As with continuous outcomes, the mixed model will estimate the cluster-level variability . Given these parameters and an assumed effect size (*β*_1_), power for the design with 100 clusters per arm and 10 children per cluster is estimated using a similar procedure as for the continuous outcome example above. Steps 1-4 and 7-8 remain the same. Steps 5 and 6 now involve simulating outcomes *y_ij _*for each child as a Bernoulli random variable with probability *p_ij _*(equation 4), and *y_ij _*is regressed on the treatment indicator *A_i_*, using a logistic regression with sandwich robust standard errors to obtain *P *values for the test  that are adjusted for clustering.

**Figure 3:**
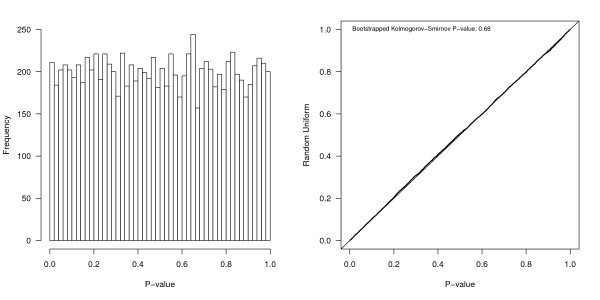
**Summary of 2-sided *P *values obtained from 10,000 simulation runs under a null treatment scenario**. The left panel includes a histogram of the *P *values and the right panel is a quantile-quantile (QQ) plot of the *P *values against a uniform random variable. The solid line in the QQ plot is the line of equality. Such diagnostic plots - using *P *values generated in a scenario where the null hypothesis is true - are useful to validate a simulation program.

After designing a simulation and implementing it in a software language, we strongly recommend setting a random number generating seed (for perfectly reproducible results) and using a simple diagnostic test to check for errors. This involves running a simulation under the null hypothesis (*β_1 _*set to zero) for a representative design scenario. Given a large enough set of simulations (e.g., 10,000), the fraction of runs in which a statistically significant difference is identified between groups should be equivalent to the Type I error rate - the probability of falsely rejecting the null hypothesis - or 5% in this case for a two-sided test (10% if using a one-sided test). A quantile-quantile plot of the empirical *P *values against the uniform distribution should fall on the line of equality; a histogram should also show a uniform distribution of the *P *values between 0 and 1 (Figure 3). The distribution of *P *values across the simulations can be tested against the uniform distribution using a bootstrapped Kolmogorov-Smirnov test [40,41].

## Results

### Comparison of Simulation and Conventional Methods

For the design described in the previous section, we compare the simulation approach for estimating study power to the more familiar analytic approach. For a continuous outcome, a design's power for a parallel cluster-randomized trial is calculated analytically as [1]:(6)

where *β *is the Type II error rate, Φ is the normal cumulative distribution function, *c *is the number of clusters per arm, *n *is the number of individuals per cluster, *d *is the mean difference between treatment groups, *σ*^2 ^is the variance of the outcome, *Z_α/2 _*is the quantile of the standard normal distribution associated with a Type I error rate of *α*, and *ρ *is the ICC (equation 2). To estimate parameters for the power calculation we use a training dataset from Indonesia that is part of an ongoing evaluation of the World Bank's Water and Sanitation Program's (WSP) Total Sanitation and Sanitation Marketing campaign [42]. The dataset includes length measurements from 2,090 children under age 24 months collected from 160 rural villages (clusters) in East Java at the baseline of the study. All length measurements (accurate to 0.1 cm) are standardized to HAZ using the WHO 2006 international standard [43]. The mean HAZ in the sample is -0.875 and its standard deviation is 1.384. To estimate the fraction of the variability explained at the village level, we estimate a mixed model regression of the form in equation 3. Estimates of the standard deviation for the village-level random effect for HAZ and the residual error are: and , respectively. This implies that the majority of the variability is at the child level, and the implied ICC is 0.482^2^/(0.482^2 ^+ 1.297^2^) = 0.12.

**Figure 4:**
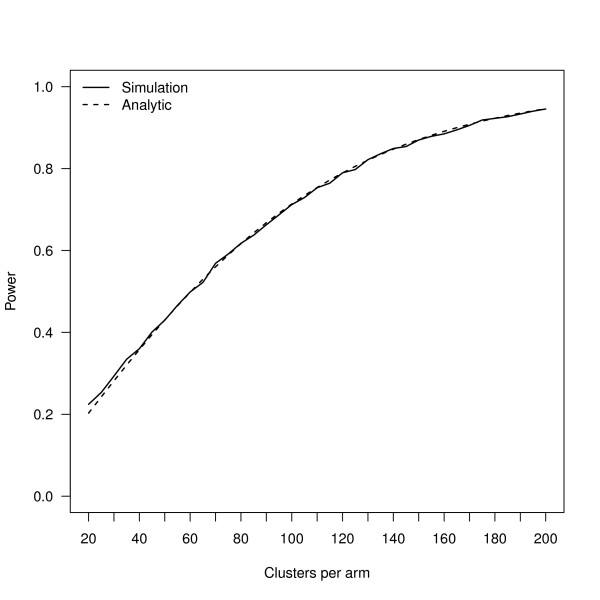
**Power curves (1-Type II error) using the simulation approach versus a conventional analytic formula for a simple cluster-randomized design described in the text**.

Given these parameter estimates, and assuming the study intervention is expected to increase mean HAZ by 0.2 SDs, we estimate power for a range of study designs with 20 children per cluster and between 20 and 200 clusters per arm. We calculate power using both the simulation approach outlined in the previous section (with 10,000 iterations per scenario) and again using equation 5. Figure 4 shows power estimates for the two approaches across the scenarios considered and demonstrates very good agreement, as expected given both are derived from the same data-generating model.

### The Use of Simulation to Estimate Power for More Complex Designs

There are many study designs for which analytic equations are not available. An example of a non-standard design is the two treatment factorial trial described in the introduction, in which sanitation mobilization is randomized at the community level, and LNS is provided to a random sub-sample of children in each village (Figure 1). We assume one child per household and that child length is measured at baseline (pre-treatment) and again two years later. This poses problems for a conventional sample size equation due to treatment at multiple levels (community and child) and correlation at multiple levels (within-community and within-child). The three hypotheses of interest include whether or not each individual intervention improves child HAZ scores on its own, and whether there is additional benefit to providing the interventions together (interaction or synergy).

Let *Y_ijt _*be the HAZ score for child *j *in village *i *at time *t*, and let *A_ijt _*be an indicator variable equal to 1 if that child has been exposed to the sanitation mobilization intervention and zero otherwise. Similarly, let *X_ijt _*be an indicator variable equal to 1 if the child is located in a household that has received nutritional supplements and zero otherwise. For the purposes of the simulation, we assume that the HAZ for each child is a function of the underlying population mean, *μ*, the treatment effects alone and in combination (*β_1_*, *β_2_*, *β_3_*), a village-level random effect *b_i_*, a child level random effect *b_ij_*, and a residual error term *ε_ijt_*:(7)

As with the simple example, we assume that the random effects and the error term are normally distributed with mean zero and are uncorrelated with each other. In this design, each child is measured twice, at baseline before the intervention (*t *= 0) and at follow-up (*t *= 1). To estimate the variability of the HAZ scores at the village and child level, we use a training dataset from a longitudinal cohort study in India, where up to two HAZ measurements are available for 1,236 children in 25 rural villages [44]. The mean (SD) of HAZ in the data is -1.98 (1.68). To estimate the cluster, child and residual variance components, we estimate a mixed-effects regression model of child HAZ on an intercept term using the training data. This is equivalent to specifying the following model for the mean HAZ:(8)

**Figure 5:**
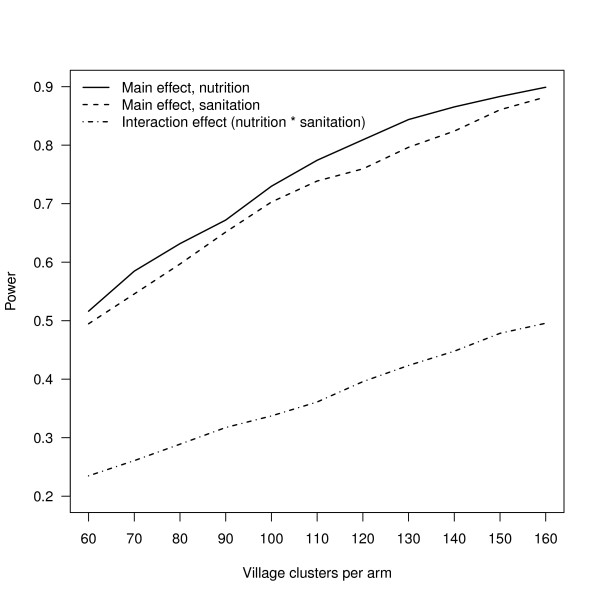
**Power curves (1-Type II error) from the two-treatment, factorial design simulation described in the text**. All three treatment effect parameters are assumed to be of equal size.

The parameter estimates obtained from the training data are:  = 0.297,  = 1.259 and  = 1.079. Using these simulation parameters, and assumed intervention effects (*β_1 _*= *β_2 _*= *β_3 _*= 0.15), we can estimate power for different design scenarios. For example, consider a design scenario where half of study villages receive the sanitation mobilization intervention, and we enroll 20 households with a child aged < 6 months in each village. Half of the study children in each village (n = 10) receive complementary LNS feeding. We assume that 10% of the children dropout between baseline and follow-up. The supporting files include code in both R and Stata to run this simulation (see additional files 1 and 2: R-programs.pdf, Stata-programs.pdf). Figure 5 plots power curves for between 60 and 160 clusters per arm using the scenario above with 10,000 iterations per run. As expected, the power to detect main effects (*β_1_*, *β_2_*) is much higher than the power to detect interaction effects (*β_3_*) of the same magnitude because the model pools information across all households, and there are twice as many children treated with either the sanitation or the LNS intervention than both combined.

Other parameters may be of interest beyond the treatment contrasts that the design implies, such as those estimated using population intervention models [45,46], where the distribution of sanitation or nutrition supplementation reflects conditions of the study population at baseline (before intervention) or of a relevant, external population. Simulation could naturally accommodate such alternate parameters of interest for which no closed form power equations exist.

## Discussion

We have demonstrated with practical examples how to use computer simulation to estimate study design power based on an assumed mixed model data generating distribution for the outcomes, which is identical to the distribution assumed for conventional power equations [1]. Simulation naturally extends conventional power equations for simple parallel trial designs by substituting programming and computer time for the effort it would require a statistician to derive analytical solutions (which for many designs may be impossible). These methods are universally applicable and can accommodate arbitrarily complex designs. The general approach can be extended to any data-generating model, and statistical test of interest (see [16] for practical examples that include the Poisson distribution, Cox-proportional hazards estimation and rank sum tests). Although we have focused on power, the process can be iterated to identify the minimum detectable effect for a fixed design.

Beyond the flexibility of simulation, an additional benefit of this approach to power studies is that it requires investigators to be more explicit about their analysis plan. The process ensures that the investigators specify a parameter of interest and estimation approach in advance, which may reduce the temptation to explore alternative modeling approaches in the presence of negative findings and is consistent with CONSORT guidelines [47]. Despite these potential benefits, we caution against the over interpretation of power simulation results. Like equation-based power calculations, the results are sensitive the assumptions about outcome variability and the data generating model (e.g., that random effects are drawn from a normal distribution), which are nearly always violated to some extent in practice. A simulation approach, like conventional power equations, will not inform investigators about optimal design choices under threats to validity like non-random losses to follow-up or systematic measurement error. We recommend the use of the diagnostic checks outlined in this article and suggest that simulations be audited in similar fashion to a primary analysis. Burton et al. [48] provide a general overview of how to conduct simulation studies in medical research. We also recommend that the characteristics of training datasets reflect the planned study population as closely as possible (e.g., age, geographic distribution, and measurement frequency).

Extensions to the basic methods in this article are possible. For example, we have used simulation to make mid-study design corrections, assuming lower levels of variability at follow-up than those observed at baseline (to reflect lower error due to improved measurement techniques). We have also used the approach to design multi-country trials where each country's cluster sizes and variance parameters differ, but a common test across countries is desired. Other extensions that involve more assumptions include more complex patterns of attrition [3], optimization using cost functions [6], or inclusion of covariates for either stratification or variance reduction [7]. For situations in which existing data are available to inform the parameters of the data generating model, one could consider adopting a Bayesian approach and simulating the posterior distribution for a design's power. This would provide a full description of estimated power, enabling the researcher to determine not just the expected power for a given design but also, for example, the probability that the power will be above an unacceptably low value.

## Conclusions

The use of simulation to estimate study design power extends conventional power equations to accommodate non-standard designs that often arise in practice. Investigators can estimate power for virtually any design as long as training datasets are available to estimate the appropriate variance parameters. The approach we have described is universally applicable for estimating the power of study designs used in epidemiologic and social science research.

## List of abbreviations used

HAZ: height-for-age Z-score; ICC: intraclass correlation coefficient; LNS: lipid-based nutrient supplementation; SD: Standard Deviation

## Competing interests

The authors declare that they have no competing interests.

## Authors' contributions

AH conceived the idea. BA and DH designed and implemented the simulations. BA, DH, JC and AH wrote the manuscript.

## Authors' information

BA is an epidemiologist in the Colford Research Group, Division of Epidemiology at the University of California, Berkeley. DH is a Ph.D. candidate in Health Policy at Harvard University. JC is Professor of Epidemiology at the University of California, Berkeley. AH is Associate Professor of Biostatistics at the University of California, Berkeley. All authors are actively involved in the design and analysis of epidemiologic field studies.

## Disclaimer

This manuscript is based on research funded in part by the Bill & Melinda Gates Foundation. The findings and conclusions contained within are those of the authors and do not necessarily reflect positions or policies of the Bill & Melinda Gates Foundation. Some of the data in this article were collected through research conducted by the Water and Sanitation Program (http://www.wsp.org). For more information, please visit http://www.wsp.org/scalingupsanitation, or send an email to wsp@worldbank.org. WSP is a multi-donor partnership created in 1978 and administered by the World Bank to support poor people in obtaining affordable, safe, and sustainable access to water and sanitation services. WSP's donors include Australia, Austria, Canada, Denmark, Finland, France, the Bill & Melinda Gates Foundation, Ireland, Luxembourg, Netherlands, Norway, Sweden, Switzerland, United Kingdom, United States, and the World Bank. The findings, interpretations, and conclusions expressed in this paper are entirely those of the authors. They do not necessarily represent the views of the Water and Sanitation Program, the World Bank and its affiliated organizations or those of the Executive Directors of the World Bank or the governments they represent.

## Pre-publication history

The pre-publication history for this paper can be accessed here:

http://www.biomedcentral.com/1471-2288/11/94/prepub

## Supplementary Material

Additional file 1 **R-programs**. R computer code used to run the simulations described in the text. Click here for file

Additional file 2 **Stata-programs**. Stata computer code used to run the simulations described in the text. Click here for file
