# Supplementary material for: Simulation methods to estimate design power: an overview for applied research
Source: BMC Med Res Methodol. 2011 Jun 20;11:94. doi: 10.1186/1471-2288-11-94 (PMC3146952; doi:10.1186/1471-2288-11-94)
Supplement: Additional file 1 — R-programs. R computer code used to run the simulations described in the text. [file 1471-2288-11-94-S1.PDF]

Example 1 in the text (standard cluster-randomized trial)

```
#Power simulation for Yij = mu + beta*Ai + bi + eij
#  (normally distributed continuous outcome with cluster (i) and residual (ij) variability)

#Function parameters:
# mu: underlying mean of the outcome in the control group
# beta: difference in outcome between treatment and control
# sdClust: standard deviation of random effect at cluster level
# sdResid: standard deviation of residual error
# nChildPerClust: number of children per cluster
# nTreatClust: number of treatment clusters
# nControlClust: number of control (untreated) clusters
# nIterations: number of iterations for simulation

rm(list=ls())
library(Hmisc)
library(rms)

fnPower <- function(mu,beta,sdClust,sdResid,nChildPerClust,nTreatClust,nControlClust,nIterations,dots=TRUE){
 start.time <- Sys.time()

 if(dots) cat("Simulations (",nIterations,") \n----|--- 1 ---|--- 2 ---|--- 3 ---|--- 4 ---| --- 5 \n",sep="")

 #objects to store pvalue, beta, and standard error from each iteration of simulation
 pVec <- betaVec <- seVec <- rep(NA,nIterations)

 #build design matrix
 nClust <- nTreatClust + nControlClust
 nObs <- nChildPerClust * nClust
 m <- matrix(NA,nrow=nObs,ncol=3)
 colnames(m) <- c("cluster","child","tx")
 m[,1] <- rep(1:nClust,each=nChildPerClust)
 m[,2] <- 1:nrow(m)
 m[,3] <- c(rep(1,nTreatClust*nChildPerClust),rep(0,nTreatClust*nChildPerClust))

 tx <- m[,3]  #treatment dummy

  for(i in 1:nIterations){

    #draw random effects
    clustRandEffect <- rep(rnorm(nClust,0,sdClust),each=nChildPerClust)
    resid <- rep(rnorm(nObs,0,sdResid))

    #create outcome
    y <- mu + beta*tx + clustRandEffect + resid

    #fit model, store p-value, beta and standard error
    o <- ols(y~tx,x=TRUE,y=TRUE)
    v <- robcov(fit=o,cluster=m[,1])
    pVec[i] <- 2*pnorm(-abs(v$coeff[2]/sqrt(diag(v$var))[2]))
    betaVec[i] <- v$coef[2]
    seVec[i] <- sqrt(diag(v$var))[2]

    if(dots) cat(".",sep="")
    if(dots && i %% 50 == 0) cat(i,"\n")

   }

   if(dots) cat("\nSimulation Run Time:",round(difftime(Sys.time(),start.time,units="hours"),3)," Hours \n")

   #calculate power
   power <- length(pVec[pVec<0.05])/length(pVec)

  return(list(power=power,p=pVec,beta=betaVec,se=seVec))
}


#Calibrate by setting beta = 0
outCalibrate <-
fnPower(mu=-0.25,beta=0,sdClust=0.482,sdResid=1.297,nChildPerClust=20,nTreatClust=50,nControlClust=50,nIterations
=10000)
```

```
outCalibrate$power
hist(outCalibrate$p)


#Create power curve for increasing number of clusters
# (here, assume equal number of clusters in treatment and control arms)
clustersPerArm <- seq(20,200,by=5)
st <- c()
for(i in 1:length(clustersPerArm)){
 st[[paste("clust",clustersPerArm[i])]] <-
fnPower(mu=0,beta=0.2,sdClust=0.482,sdResid=1.297,nChildPerClust=20,nTreatClust=clustersPerArm[i],nControlClust=c
lustersPerArm[i],nIterations=10000)
}
powerVals <- lapply(st,function(x) x[[1]])
plot(clustersPerArm,unlist(powerVals),type='l',ylab='Power',xlab="Clusters per arm",ylim=c(0,1))
```

Example 2 in the text: 2-treatment, 2-level factorial trial

```
#Power by simulation for a normally distributed continuous outcome with cluster, child and residual variability
# yij = mu + beta1*A1it + beta2*A2ijt + beta3*A1A2ijt + bi + bij + eijt

#Population parameters
#mu: underlying mean of the outcome in the control group
#beta1: effect size of treatment 1 (assigned at village level)
#beta2: effect size of treatment 2 (cross-cut intervention assigned at child (household) level)
#beta3: effect size of interaction of treatment 1 and treatment 2
#sdClust: sd of random effect at the cluster level (sd for the bi)
#sdChild: sd of random effect at the child level (sd for the bij)
#sdResid: sd of residual error (sd for the eij)

#Design parameters
#nChildPerClust: number of children per cluster
#nTreatClust: number of clusters receiving treatment 1
#nControlClust: number of clusters not receiving treatment 1
#nTx2perClust: number of children to receive treatment 2 per cluster
#dropout: fraction of post-baseline observations lost to follow-up

#nIterations: number of iterations in simulation



rm(list=ls())
library(Hmisc)
library(rms)

fnPower <-
function(mu,beta1,beta2,beta3,sdClust,sdChild,sdResid,nChildPerClust,nTreatClust,nControlClust,nTx2perClust,drop
out,nIterations,dots=TRUE){
 start.time <- Sys.time()

 if(dots) cat("Simulations (",nIterations,") \n----|--- 1 ---|--- 2 ---|--- 3 ---|--- 4 ---| --- 5 \n",sep="")

 if(nTx2perClust>nChildPerClust){
  print("There are not enough children per cluster for your selected value of nTx2perClust")
  return(NULL)
 }

 #objects to store pvalue, beta, and standard error from each iteration of simulation
 pVals <- betaVals <- seVals <- matrix(NA,nrow=nIterations,ncol=3)

 #build design matrices
 nClust <- nTreatClust + nControlClust
 nObsPreTx <- nChildPerClust * nClust
 nObsPostTx <- nChildPerClust * nClust
 nObs <- nObsPreTx+ nObsPostTx
 nChild <- nClust * nChildPerClust

 #pre-treatment
 mPre <- matrix(NA,nrow=nObsPreTx,ncol=6)
 colnames(mPre) <- c("cluster","child","postObs","tx1","tx2","tx12")
 mPre[,1] <- rep(1:nClust,each=nChildPerClust)
 mPre[,2] <- 1:(nChildPerClust * nClust)
 mPre[,3:6] <- 0

 #post-treatment
 mPost <- matrix(NA,nrow=nObsPostTx,ncol=6)
 colnames(mPost) <- c("cluster","child","postObs","tx1","tx2","tx12")
 mPost[,1] <- rep(1:nClust,each=nChildPerClust)
 mPost[,2] <- 1:(nChildPerClust*nClust)
 mPost[,3] <- 1
 mPost[,4] <- c(rep(1,nTreatClust*nChildPerClust),rep(0,nControlClust*nChildPerClust))
 mPost[,5] <- rep(c(rep(1,nTx2perClust),rep(0,(nChildPerClust-nTx2perClust))),nClust)
 mPost[,6] <- mPost[,4]*mPost[,5]

 #allow for dropout before post intervention (assumes no follow-up visits for child who dropped out)
 if(dropout>0){
  keep <- sample(1:(nChildPerClust*nClust),round((nChildPerClust*nClust)*(1-dropout)))
```

```
  mPost <- mPost[mPost[,2] %in% keep,]
 }

  #now combine
 m    <- rbind(mPre,mPost)
 tx1  <- m[,4]
 tx2  <- m[,5]
 tx12 <- m[,6]

  for(i in 1:nIterations){

    #draw random effects
    clustRE <- rnorm(nClust,0,sdClust)
    childRE <- rnorm(nChild,0,sdChild)
    residRE <- rnorm(nrow(m),0,sdResid)

    #create outcome
    y <- mu + beta1*tx1 + beta2*tx2 + beta3*tx12 + clustRE[m[,1]] + childRE[m[,2]] + residRE

    #fit model, store p-value, beta and standard error
    o <- ols(y~tx1+tx2+tx12,x=TRUE,y=TRUE)
    v <- robcov(fit=o,cluster=m[,1])
    pVals[i,] <- 2*pnorm(-abs(v$coeff[2:4]/sqrt(diag(v$var))[2:4]))
    betaVals[i,] <- v$coef[2:4]
    seVals[i,] <- sqrt(diag(v$var))[2:4]

    if(dots) cat(".",sep="")
    if(dots && i %% 50 == 0) cat(i,"\n")

   }

   if(dots) cat("\nSimulation Run Time:",round(difftime(Sys.time(),start.time,units="hours"),3)," Hours \n")

   #calculate power
   powerOut <- apply(pVals,2,function(x) length(x[x<0.05])/length(x))

   return(list(power=powerOut,p=pVals,beta=betaVals,se=seVals))
 }

#calibrate by setting betas = 0
outCalibrate  <-
fnPower(mu=-1,beta1=0,beta2=0,beta3=0,sdClust=0.297,sdChild=1.259,sdResid=1.079,nChildPerClust=10,nTreatClust=50
,nControlClust=50,nTx2perClust=5,dropout=0,nIterations=10000,dots=TRUE)
outCalibrate$power
par(mfrow=c(2,2))
hist(outCalibrate$p[,1],main="beta1",xlab="p value")
hist(outCalibrate$p[,2],main="beta2",xlab="p value")
hist(outCalibrate$p[,3],main="beta3",xlab="p value")

#create power curve for increasing number of clusters
clustersPerArm <- seq(60,160,10)

st <- c()
for(i in 1:length(clustersPerArm)){
 st[[paste("clust",clustersPerArm[i])]] <-
fnPower(mu=-1.98,beta1=0.15,beta2=0.15,beta3=0.15,sdClust=0.297,sdChild=1.259,sdResid=1.079,nChildPerClust=20,nT
reatClust=clustersPerArm[i],nControlClust=clustersPerArm[i],nTx2perClust=10,dropout=0.1,nIterations=10000,dots=T
RUE)
}
powerVals <- lapply(st,function(x) x[[1]])

plot(clustersPerArm,unlist(lapply(powerVals,function(x) x[[1]])),type='l',ylab='Power',xlab="Village clusters
per arm",ylim=c(0,1),lty=2)
lines(clustersPerArm,unlist(lapply(powerVals,function(x) x[[2]])),lty=1)
lines(clustersPerArm,unlist(lapply(powerVals,function(x) x[[3]])),lty=3)
legend(x=min(clustersPerArm),y=1.05,legend=c("Main effect, household treatment","Main effect, village
treatment","Interaction effect"),lty=c(1,2,3),bty='n',cex=0.8)
```

Example 1 in the text with an alternate, binary outcome

```
#Power simulation for Yij ~ [1 + exp(-[mu + beta*Ai + bi])]^-1
#  (binary outcome  with cluster (i) variability)

# NOTE: THIS SIMULATION IS NOT PRESENTED IN THE TEXT, BUT IS ANALOGOUS TO THE
# SIMPLE CLUSTER-RANDOMIZED TRIAL WITH A CONTINOUS OUTCOME (EXAMPLE 1).
# HERE, THE OUTCOME IS SIMULATED AS BINARY (AS AN EXAMPLE).

#Function parameters:
# mu: mean prevalence of outcome in the control group
# beta: odds ratio of outcome in treatment vs. control
# sdClust: standard deviation of random effect at cluster level
# nChildPerClust: number of children per cluster
# nTreatClust: number of treatment clusters
# nControlClust: number of control (untreated) clusters
# nIterations: number of iterations for simulation


rm(list=ls())
library(Hmisc)
library(rms)


#Function for calculating study power for given design and treatment effects
fnPower <- function(mu,beta,sdClust,nChildPerClust,nTreatClust,nControlClust,nIterations,dots=TRUE){
 start.time <- Sys.time()

 if(dots) cat("Simulations (",nIterations,") \n----|--- 1 ---|--- 2 ---|--- 3 ---|--- 4 ---| --- 5 \n",sep="")

 #objects to store pvalue, beta, and standard error from each iteration of simulation
 pVec <- betaVec <- seVec <- rep(NA,nIterations)

 #build design matrix
 nClust <- nTreatClust + nControlClust
 nObs <- nChildPerClust * nClust
 m <- matrix(NA,nrow=nObs,ncol=3)
 colnames(m) <- c("cluster","child","tx")
 m[,1] <- rep(1:nClust,each=nChildPerClust)
 m[,2] <- 1:nrow(m)
 m[,3] <- c(rep(1,nTreatClust*nChildPerClust),rep(0,nTreatClust*nChildPerClust))

 tx <- m[,3]  #treatment dummy

  for(i in 1:nIterations){

    #draw random effects for clusters
    clustRandEffect <- rep(rnorm(nClust,0,sdClust),each=nChildPerClust)

    #create outcome
    prob <- 1/(1 + exp(-(log(mu/(1-mu)) + log(beta)*tx + clustRandEffect)))
    y <- rbinom(nObs,1,prob)

    #fit model, store p-value, beta and standard error
    o <- try(lrm(y~tx,x=TRUE,y=TRUE))

    #lrm() function occasionally gets stuck in null scenario
    # (if it does, change initial parameter value as work around)
    #Note: We have contacted author of rms package, who is correcting this error
    # (i.e., we will be able to remove this line before final publication)
    if(inherits(o,"try-error")==TRUE){
     o <- lrm(y~tx,x=TRUE,y=TRUE,initial=0.5)
    }

    v <- robcov(fit=o,cluster=m[,1])
    pVec[i] <- 2*pnorm(-abs(v$coeff[2]/sqrt(diag(v$var))[2]))
    betaVec[i] <- v$coef[2]
    seVec[i] <- sqrt(diag(v$var))[2]

    if(dots) cat(".",sep="")
    if(dots && i %% 50 == 0) cat(i,"\n")
```

```
  }

  if(dots) cat("\nSimulation Run Time:",round(difftime(Sys.time(),start.time,units="hours"),3)," Hours \n")

  #calculate power
  power <- length(pVec[pVec<0.05])/length(pVec)

  return(list(power=power,p=pVec,beta=betaVec,se=seVec))
 }


#Calibrate by setting beta = 0
outCalibrate <-
fnPower(mu=0.1,beta=1,sdClust=0.8,nChildPerClust=20,nTreatClust=50,nControlClust=50,nIterations=10000)
outCalibrate$power
hist(outCalibrate$p)



#Generate power power curve for different number of clusters per arm (with 20 children per cluster)
# (here, assume same number of clusters in treatment and control arms)
clustersPerArm <- seq(20,200,by=5)
st <- c()
for(i in 1:length(clustersPerArm)){
 st[[paste("clust",clustersPerArm[i])]] <-
fnPower(mu=0.1,beta=0.8,sdClust=0.8,nChildPerClust=20,nTreatClust=clustersPerArm[i],nControlClust=clustersPerArm[
i],nIterations=10000)
}
powerVals <- lapply(st,function(x) x[[1]])
plot(clustersPerArm,unlist(powerVals),type='l',ylab="Power",xlab="Clusters per arm",ylim=c(0,1))
```

Example 1 in the text : Analytic power calculation

```
#-----------------------------------------
# Program:       1-analyticpower.R
# description :
#
# estimate sample size for clustered
# data with a continuous outcome.
# Derived on page 361 of Murray 1998
# Design and Analysis of Group Randomized Trials
#
#-----------------------------------------

rm(list=ls())

#-----------------------------------------
# sample size function (est. number of groups)
#-----------------------------------------
sampsi <- function(d,sd,r,n,alpha=0.05,beta=0.2) {
    # d  effect size (difference)
    # sd standard deviation of the outcome
    # n  number of individuals per group
    # r  ICC for measures
    # alpha type I error
    # beta type II error
    za <- qnorm(1-(alpha/2))
    zb <- qnorm(1-beta)
    a  <- (za + zb)^2
    b  <- 2*(sd^2)
    c  <- 1 + (n-1)*r
    d  <- n*(d^2)
    return(  ceiling(  a*b*c*(1/d) )  )
}

#-----------------------------------------
# power function (est. power)
# re-arranged version of sampsi
#-----------------------------------------
cpow <- function(c,n,d,sd,r,alpha=0.05) {
    # d  effect size (difference)
    # sd standard deviation of the outcome
    # n  number of individuals per group
    # c  number of groups (clusters)
    # r  ICC for measures
    # alpha type I error
    za <- qnorm(1-(alpha/2))
    a  <- c*n*(d^2)
    b  <- 2*(sd^2)
    c  <- 1 + (n-1)*r
    return(  pnorm( sqrt(a/(b*c)) - za ) )
}

# reverse the two functions:
# (power not exactly 0.8 due to rounding from ceiling function)
cpow(d=0.117,sd=0.48,r=0.03,n=50,c=sampsi(d=0.117,sd=0.48,r=0.03,n=50))


#-----------------------------------------
# Calculate power for different designs
#
# columns of the table: children/cluster
# rows of the clusters
#-----------------------------------------
# parameters from Indonesia dataset
# sd(Y) = 1.384
#   ICC = var(cluster) / [var(cluster) + var(residual)]
#       = 0.232 / ( 0.232 + 1.682) = 0.12

v.cpow <- Vectorize(cpow,c("c","n"),SIMPLIFY=FALSE)
n <- c(20)
```

```
c <- seq(20,200,by=5)

pow <- outer(c,n,v.cpow,d=0.2,sd=1.384,r=0.12)
rownames(pow) <- c
colnames(pow) <- n

# save output for plotting
save.image(file="1-analyticpower.RData")
```
