# Supplementary material for: Simulation methods to estimate design power: an overview for applied research
Source: BMC Med Res Methodol. 2011 Jun 20;11:94. doi: 10.1186/1471-2288-11-94 (PMC3146952; doi:10.1186/1471-2288-11-94)
Supplement: Additional file 2 — Stata-programs. Stata computer code used to run the simulations described in the text. [file 1471-2288-11-94-S2.PDF]

Examples of calculating variance parameters with mixed models (continuous and binary outcomes)

```
  ___  ____  ____  ____  ____ tm
 /__    /   ____/   /   ____/
___/   /   /___/   /   /___/   10.1   Copyright 1984-2009
  Statistics/Data Analysis            StataCorp
                                      4905 Lakeway Drive
                                      College Station, Texas 77845 USA
                                      800-STATA-PC        http://www.stata.com
                                      979-696-4600        stata@stata.com
                                      979-696-4601 (fax)

Notes:
      1.  100.00 MB allocated to data
      2.  Stata running in batch mode

. do 2-simpower-varests.do

. capture log close

. set more off

. clear mata

. clear

.
. *--------------------------------------------
. * Program:             2-simpower-varests.do
. * Description:
. *
. * Estimate cluster  level variance
. * in Height-for-age Z-scores and diarrhea
. * using multiple datasets
. *--------------------------------------------
.
.
. *--------------------------------------------
. * Input Files:
. *       IndoAnthro.dta
. *       trichy_anthro.dta
. *
. * Output Files:
. *       (none)
. *--------------------------------------------
.
. set mem 1000m
(1024000k)

.
.
. *------------------------------------------
. * WSP Baseline Indonesia Data
. *------------------------------------------
.
. use ~/dropbox/wsp/indonesia/data/final/IndoAnalysis, clear
.
. * drop outliers
. drop if fzhgt == 1
(4 observations deleted)
.
. * create individual ID
. gen child = idhh1*100*100 + idhh2*100 + idindiv
(837 missing values generated)
.
. * means
. sum zhgt, d

                Length/height-for-age z-score
-------------------------------------------------------------
      Percentiles      Smallest
 1%        -4.09             -6
 5%        -3.12          -5.46
10%        -2.59          -4.97       Obs                2090
25%        -1.83           -4.8       Sum of Wgt.        2090

50%         -.91                      Mean          -.8782679
                        Largest       Std. Dev.      1.385462
75%            0           3.55
90%          .86            3.6       Variance       1.919504
95%         1.45           3.83       Skewness       .1252677
99%         2.67           4.26       Kurtosis       3.273667

. sum diar7d, d

                  Diarrhea in prev. 7 days
-------------------------------------------------------------
      Percentiles      Smallest
 1%            0              0
 5%            0              0
10%            0              0       Obs                2340
25%            0              0       Sum of Wgt.        2340

50%            0                      Mean           .0726496
```

```
                        Largest       Std. Dev.      .2596159
75%           0             1
90%           0             1       Variance       .0674004
95%           1             1       Skewness       3.292878
99%           1             1       Kurtosis       11.84305

.
. * ICC
. loneway zhgt idhh1

      One-way Analysis of Variance for zhgt: Length/height-for-age z-score

                                              Number of obs =      2090
                                                  R-squared =    0.1870

    Source                SS         df      MS            F     Prob > F
-------------------------------------------------------------------------
Between idhh1          749.9298    159    4.7165396      2.79     0.0000
Within idhh1          3259.9149   1930    1.6890751
-------------------------------------------------------------------------
Total                 4009.8447   2089    1.9195044

         Intraclass       Asy.
         correlation      S.E.       [95% Conf. Interval]
         ------------------------------------------------
            0.12068     0.01943       0.08261     0.15876

         Estimated SD of idhh1 effect            .4814793
         Estimated SD within idhh1               1.299644
         Est. reliability of a idhh1 mean         0.64188
              (evaluated at n=13.06)

. loneway diar7d idhh1

       One-way Analysis of Variance for diar7d: Diarrhea in prev. 7 days

                                              Number of obs =      2340
                                                  R-squared =    0.1086

    Source                SS         df      MS            F     Prob > F
-------------------------------------------------------------------------
Between idhh1         17.120461    159    .10767585      1.67     0.0000
Within idhh1          140.52911   2180     .0644629
-------------------------------------------------------------------------
Total                 157.64957   2339    .06740042

         Intraclass       Asy.
         correlation      S.E.       [95% Conf. Interval]
         ------------------------------------------------
            0.04384     0.01217       0.01999     0.06768

         Estimated SD of idhh1 effect            .0543637
         Estimated SD within idhh1               .2538954
         Est. reliability of a idhh1 mean         0.40132
              (evaluated at n=14.62)

.
. * cluster level variability
. xtmixed zhgt || idhh1:

Performing EM optimization:

Performing gradient-based optimization:

Iteration 0:   log restricted-likelihood =  -3595.343
Iteration 1:   log restricted-likelihood =  -3595.343

Computing standard errors:

Mixed-effects REML regression                   Number of obs      =      2090
Group variable: idhh1                           Number of groups   =       160

                                                Obs per group: min =         3
                                                               avg =      13.1
                                                               max =        20


                                                Wald chi2(0)       =         .
Log restricted-likelihood =  -3595.343          Prob > chi2        =         .

------------------------------------------------------------------------------
        zhgt |      Coef.   Std. Err.      z    P>|z|     [95% Conf. Interval]
-------------+----------------------------------------------------------------
       _cons |  -.8755489   .0477322   -18.34   0.000    -.9691023   -.7819956
------------------------------------------------------------------------------

------------------------------------------------------------------------------
  Random-effects Parameters  |   Estimate   Std. Err.     [95% Conf. Interval]
-----------------------------+------------------------------------------------
idhh1: Identity              |
                   sd(_cons) |   .4809685   .0421668      .4050338    .5711393
-----------------------------+------------------------------------------------
                sd(Residual) |   1.299009   .0208865      1.258711    1.340598
```

```
------------------------------------------------------------------------------
LR test vs. linear regression: chibar2(01) =   107.45 Prob >= chibar2 = 0.0000

. xtmelogit diar7d || idhh1:

Refining starting values:

Iteration 0:   log likelihood =  -605.2137
Iteration 1:   log likelihood = -603.08229
Iteration 2:   log likelihood = -599.49114

Performing gradient-based optimization:

Iteration 0:   log likelihood = -599.49114
Iteration 1:   log likelihood = -599.24394
Iteration 2:   log likelihood = -599.24144
Iteration 3:   log likelihood = -599.24144

Mixed-effects logistic regression               Number of obs      =      2340
Group variable: idhh1                           Number of groups   =       160

                                                Obs per group: min =         3
                                                               avg =      14.6
                                                               max =        22

Integration points =   7                        Wald chi2(0)       =         .
Log likelihood = -599.24144                     Prob > chi2        =         .

------------------------------------------------------------------------------
      diar7d |      Coef.   Std. Err.      z    P>|z|     [95% Conf. Interval]
-------------+----------------------------------------------------------------
       _cons |  -2.802107   .1280623   -21.88   0.000    -3.053104   -2.551109
------------------------------------------------------------------------------

------------------------------------------------------------------------------
  Random-effects Parameters  |   Estimate   Std. Err.     [95% Conf. Interval]
-----------------------------+------------------------------------------------
idhh1: Identity              |
                   sd(_cons) |   .7890793   .1336887      .5661181    1.099852
------------------------------------------------------------------------------
LR test vs. logistic regression: chibar2(01) =    20.37 Prob>=chibar2 = 0.0000

.
.
. *------------------------------------------
. * Tamil Nadu Data
. *------------------------------------------
. use ~/dropbox/trichy/data/fielddata/final/trichy_anthro, clear
.
. * drop outliers
. drop if zhgt <-6 | zhgt > 6
(292 observations deleted)
.
. * mean
. sum zhgt, d

                       Z-score, height
-------------------------------------------------------------
      Percentiles      Smallest
 1%        -5.71          -5.99
 5%        -4.64          -5.98
10%        -3.98          -5.97       Obs                1969
25%        -3.02          -5.96       Sum of Wgt.        1969

50%        -2.03                      Mean          -1.979822
                        Largest       Std. Dev.       1.68488
75%        -1.07           5.36
90%          .03           5.49       Variance       2.838821
95%          .89           5.53       Skewness       .6607083
99%         3.05           5.72       Kurtosis       4.714499

.
. * ICC
. loneway zhgt vilid

             One-way Analysis of Variance for zhgt: Z-score, height

                                              Number of obs =      1969
                                                  R-squared =    0.0477

    Source                SS         df      MS            F     Prob > F
-------------------------------------------------------------------------
Between vilid          266.32439     24     11.09685      4.05     0.0000
Within vilid           5320.4752   1944      2.73687
-------------------------------------------------------------------------
Total                  5586.7996   1968     2.838821

         Intraclass       Asy.
         correlation      S.E.       [95% Conf. Interval]
         ------------------------------------------------
            0.03749     0.01430       0.00947     0.06551

         Estimated SD of vilid effect            .3264965
```

```
         Estimated SD within vilid               1.654349
         Est. reliability of a vilid mean         0.75337
              (evaluated at n=78.42)

.
. * cluster level variability
. xtmixed zhgt || vilid:

Performing EM optimization:

Performing gradient-based optimization:

Iteration 0:   log restricted-likelihood = -3802.1756
Iteration 1:   log restricted-likelihood = -3802.1756

Computing standard errors:

Mixed-effects REML regression                   Number of obs      =      1969
Group variable: vilid                           Number of groups   =        25

                                                Obs per group: min =        28
                                                               avg =      78.8
                                                               max =       117


                                                Wald chi2(0)       =         .
Log restricted-likelihood = -3802.1756          Prob > chi2        =         .

------------------------------------------------------------------------------
        zhgt |      Coef.   Std. Err.      z    P>|z|     [95% Conf. Interval]
-------------+----------------------------------------------------------------
       _cons |  -1.955312   .0738645   -26.47   0.000    -2.100083    -1.81054
------------------------------------------------------------------------------

------------------------------------------------------------------------------
  Random-effects Parameters  |   Estimate   Std. Err.     [95% Conf. Interval]
-----------------------------+------------------------------------------------
vilid: Identity              |
                   sd(_cons) |   .3138513   .0600528      .2157017    .4566613
-----------------------------+------------------------------------------------
                sd(Residual) |   1.653899   .0265105      1.602747    1.706683
------------------------------------------------------------------------------
LR test vs. linear regression: chibar2(01) =    41.57 Prob >= chibar2 = 0.0000

.
. * cluster and child level variance
. xtmixed zhgt || vilid: || individ:

Performing EM optimization:

Performing gradient-based optimization:

Iteration 0:   log restricted-likelihood = -3654.9568
Iteration 1:   log restricted-likelihood = -3654.9462
Iteration 2:   log restricted-likelihood = -3654.9462

Computing standard errors:

Mixed-effects REML regression                   Number of obs      =      1969

-----------------------------------------------------------
                |   No. of       Observations per Group
 Group Variable |   Groups    Minimum    Average    Maximum
----------------+------------------------------------------
          vilid |       25         28       78.8        117
        individ |     1236          1        1.6          2
-----------------------------------------------------------

                                                Wald chi2(0)       =         .
Log restricted-likelihood = -3654.9462          Prob > chi2        =         .

------------------------------------------------------------------------------
        zhgt |      Coef.   Std. Err.      z    P>|z|     [95% Conf. Interval]
-------------+----------------------------------------------------------------
       _cons |  -1.982695   .0747916   -26.51   0.000    -2.129284   -1.836107
------------------------------------------------------------------------------

------------------------------------------------------------------------------
  Random-effects Parameters  |   Estimate   Std. Err.     [95% Conf. Interval]
-----------------------------+------------------------------------------------
vilid: Identity              |
                   sd(_cons) |   .2968767   .0643702      .1940951    .4540855
-----------------------------+------------------------------------------------
individ: Identity            |
                   sd(_cons) |   1.259798   .0410206      1.181911    1.342818
-----------------------------+------------------------------------------------
                sd(Residual) |   1.078078   .0278651      1.024824      1.1341
------------------------------------------------------------------------------
LR test vs. linear regression:       chi2(2) =   336.02   Prob > chi2 = 0.0000

Note: LR test is conservative and provided only for reference.

.
```

Example 1 in the text (standard cluster-randomized trial)

```
capture log close
set more off
clear mata
clear

*--------------------------------------------
* simpower-ex1-ols.do
    * Power simulation for Yij ~ mu + beta*Ai + bi + eij
            * (continuous outcome with cluster (i) and residual (ij) variability)
            * parameters:
            * tclust   : number of treatment clusters
            * cclust   : number of comparison clusters
            * nchild   : number of children per cluster
            * mu       : underlying mean of the outcome in the control group
            * sdclust  : sd of random effect at the cluster level
            * sdresid  : sd of residual error
            * diff     : difference due to the treatment
            * onesided : logical (one-sided test? default is two-sided)
*--------------------------------------------
capture program drop simpowerex1
program define simpowerex1, rclass
            version 9.0
            syntax [, tclust(real 100) cclust(real 100) nchild(real 20) mu(real 0) sdchild(real 0) sdclust(real
0.1) sdresid(real 1) diff(real 0)  onesided]
* internal calculations
            local nclust   = `tclust'+`cclust'   /* total num clusters */
            local totobs   = `nchild'*`nclust'   /* total observations */
            local trobs    = `nchild'*`tclust'   /* total treatment observations */
            local obspercl = `nchild'            /* observations per cluster */
            if("`onesided'"=="onesided") local tail = 1
                        else local tail = 2
            local matsize  = `obspercl'+10
            set mat `matsize'
* create a child level dataset
            set obs `totobs'
            gen obsnum  = _n
            gen byte _x = 1 if mod(obsnum[_n-1],`obspercl')==0
            gen clustid = sum(_x)
* generate random effects for clusters
            sort clustid
            by   clustid: gen _rcl = invnormal(uniform())*`sdclust' if _n==1
            by   clustid: egen randclust = max(_rcl)
* assign treatment
            gen byte tr = (obsnum <= (`trobs'))
* simulate outcome
            gen double y = `mu' + randclust  + `diff'*tr + invnormal(uniform())*`sdresid'
* run model and return results
            regress y tr, cluster(clustid) robust
            return scalar beta = _b[tr]
            return scalar p    = `tail'*normal(-abs(_b[tr]/_se[tr]))
            drop _all
end simpowerex1

*--------------------------------------------
* Example : code check with null scenarios
* (should get ~5% power -- Type I error - due to two-sided p-value)
*--------------------------------------------
set seed 1978
clear
#delimit;
simulate beta=r(beta) p=r(p), reps(10000): simpowerex1,
tclust(100) cclust(100) nchild(10) mu(0) sdclust(0.3) sdresid(1) diff(0);
#delimit cr
gen p05 = p<0.05
sum p05


*--------------------------------------------
* run simulations for cluster sizes 20(5)200,
* with 20 children per cluster
*--------------------------------------------
```

```
* open a postfile to store results
tempname memhold
tempfile results
postfile `memhold' clust child p using `results'

local nlist "20"
local clist "20(5)200"
foreach n of numlist `nlist' {
            foreach c of numlist `clist' {
                        clear
                        #delimit;
                        simulate beta=r(beta) p=r(p), reps(10000): simpowerex1,
                        tclust(`c') cclust(`c') nchild(`n') mu(0) sdclust(0.482) sdresid(1.297) diff(0.2);
                        #delimit cr
                        gen p05 = p<0.05
                        di as res _n "POWER FOR `n' CHILDREN PER CLUSTER, CLUSTER SIZE `c'"
                        sum p05
                        qui sum p05, meanonly
                        local p = r(mean)
                        post `memhold' (`c') (`n') (`p')
            }
}

postclose `memhold'
use `results', clear
outsheet using "~/dropbox/powersim/output/simpower-ex1.csv", comma replace


exit
```

Example 2 in the text: 2-treatment, 2-level factorial trial

```
capture log close
set more off
clear mata
clear


*--------------------------------------------
* simpower-ex2.do
* Power simulation for Yijt ~ mu + b1*A1it + b2*A2ijt + b3*A1A2ijt + bi + bij + eijt
* power simulation for a continuous outcome with cluster (i), child (ij) and residual (ijt) variability
* two treatments: tr 1 (cluster level) and tr 2 (child level)
* allows for multiple visits (baseline + follow-up)
* parameters   :
            * tclust   : number of treatment clusters, treatment 1 (cluster level)
            * cclust   : number of comparison clusters
            * nchild   : number of children per cluster
            * t2frac   : proportion of children treated with treatment 2 (cross-cut child level intervention)
            * bvisit   : number of baseline (pre-intervention) measurements
            * fvisit   : number of follow-up (post-intervention) measurements
            * mu       : underlying mean of the outcome in the control group
            * sdchild  : sd of random effect at the child level
            * sdclust  : sd of random effect at the cluster level
            * sdresid  : sd of residual error
            * b1       : difference due to treatment 1 (cluster level)
            * b2       : difference due to treatment 2 (child level)
            * b3       : difference due to treatment 1 and 2 combined
            * dropout  : proportion of post-baseline observations lost to follow-up
            * onesided : logical (one-sided test? default is two-sided)
            * (returned values: p1, p2 and p3 are the p-values for each coefficient)
*--------------------------------------------

capture program drop simpowerex2
program define simpowerex2, rclass
            version 9.0
            syntax [, tclust(real 100) cclust(real 100) nchild(real 20) tr2frac(real 0.5) bvisit(real 1) fvisit(real 1)  mu(real 0)
sdchild(real 0) sdclust(real 0.1) sdresid(real 1) b1(real 0) b2(real 0) b3(real 0) dropout(real 0) onesided ]
* internal calculations
            local nvisit   = `bvisit'+`fvisit'            /* total num visits */
            local nclust   = `tclust'+`cclust'            /* total num clusters */
            local totobs   = `nvisit'*`nchild'*`nclust'   /* total observations */
            local tr1obs   = `nvisit'*`nchild'*`tclust'   /* total treatment 1 observations */
            local obspercl = `nvisit'*`nchild'            /* observations per cluster */
            local tr2obs   = `obspercl'*(`tr2frac')       /* treatment 2 observations per cluster */
            if("`onesided'"=="onesided") local tail = 1
                        else local tail = 2
            local matsize  = `obspercl'+10
            set mat `matsize'
* create a child-visit level dataset
            set obs `totobs'
            gen obsnum = _n
            gen byte _x = 1 if mod(obsnum[_n-1],`obspercl')==0
            gen byte _y = 1 if mod(obsnum[_n-1],`nvisit')==0
            gen clustid = sum(_x)
            gen childid = sum(_y)
            bysort clustid childid: gen visit = _n
* generate random effects for clusters & children
            sort clustid
            by clustid: gen _rcl = invnormal(uniform())*`sdclust' if _n==1
            by clustid: egen randclust = max(_rcl)
            sort childid
            by childid: gen _rch = invnormal(uniform())*`sdchild' if _n==1
            by childid: egen randchild = max(_rch)
* assign treatments
            gen byte tr1 = (obsnum <= (`tr1obs')) & (visit > `bvisit')
            bysort clustid: gen byte tr2 = (_n <= (`tr2obs')) & (visit > `bvisit')
            gen byte tr12 = tr1*tr2
* simulate outcome
            gen double y = `mu' + randclust + randchild + `b1'*tr1 + `b2'*tr2 + `b3'*tr12 + invnormal(uniform())*`sdresid'
* account for dropout
            gen double u = uniform()
            drop if (u <= `dropout') & (visit > `bvisit')
* run model and return results
            regress y tr1 tr2 tr12, cluster(clustid) robust
            return scalar beta1 = _b[tr1]
            return scalar beta2 = _b[tr2]
            return scalar beta3 = _b[tr12]
            return scalar p1    = `tail'*normal(-abs(_b[tr1]/_se[tr1]))
            return scalar p2    = `tail'*normal(-abs(_b[tr2]/_se[tr2]))
            return scalar p3    = `tail'*normal(-abs(_b[tr12]/_se[tr12]))
            drop _all
end simpowerex2


*--------------------------------------------
* Example : code check with null scenarios
* (should get ~5% power -- Type I error - due to two-sided p-value)
```

```
*--------------------------------------------

set more off
set seed 1978
#delimit;
simulate beta1=r(beta1) beta2=r(beta2) beta3=r(beta3) p1=r(p1) p2=r(p2) p3=r(p3), reps(10000): simpowerex2,
tclust(100) cclust(100) tr2frac(0.5) nchild(20) bvisit(1) fvisit(1)
mu(0) sdclust(0.297) sdchild(1.259) sdresid(1.079)
b1(0) b2(0) b3(0) dropout(0);
#delimit cr
gen p1_05 = p1<0.05
gen p2_05 = p2<0.05
gen p3_05 = p3<0.05
sum *_05

* output the results to a file for plotting
outsheet using "~/dropbox/powersim/output/simpower-ex2-codecheck.csv", comma replace


*--------------------------------------------
* run simulations for clusters per arm 60(10)160,
* with 20 children per cluster
* 1 baseline visit, 1 follow-up visit
* B1 = B2 = B3 = 0.15
* 10% dropout after baseline
*--------------------------------------------

* open a postfile to store results
tempname memhold
tempfile results
postfile `memhold' clust child p1 p2 p3 using `results'

set seed 19780330
local nlist "20"
local clist "60(10)160"
foreach n of numlist `nlist' {
            foreach c of numlist `clist' {

                        * run the simulation
                        clear
                        #delimit;
                        simulate
                                    beta1=r(beta1) beta2=r(beta2) beta3=r(beta3)
                                    p1=r(p1) p2=r(p2) p3=r(p3), reps(10000):
                        simpowerex2,
                                    tclust(`c') cclust(`c') tr2frac(0.5) nchild(`n')
                                    mu(-1.98) sdclust(0.297) sdchild(1.259) sdresid(1.079)
                                    bvisit(1) fvisit(1) b1(0.15) b2(0.15) b3(0.15) dropout(0.1);
                        #delimit cr

                        * summarize power
                        gen p1_05 = p1<0.05
                        gen p2_05 = p2<0.05
                        gen p3_05 = p3<0.05
                        di as res _n "POWER FOR `n' CHILDREN PER CLUSTER, `c' CLUSTERS PER ARM"
                        sum *_05

                        qui sum p1_05, meanonly
                        local p1 = r(mean)
                        qui sum p2_05, meanonly
                        local p2 = r(mean)
                        qui sum p3_05, meanonly
                        local p3 = r(mean)

                        post `memhold' (`c') (`n') (`p1') (`p2') (`p3')

            }
}
postclose `memhold'
use `results', clear
outsheet using "~/dropbox/powersim/output/simpower-ex2.csv", comma replace


exit
```

Example 1 in the text with an alternate, binary outcome

```
capture log close
set more off
clear mata
clear

* NOTE: THIS SIMULATION IS NOT PRESENTED IN THE TEXT, BUT IS ANALOGOUS TO THE
* SIMPLE CLUSTER-RANDOMIZED TRIAL WITH A CONTINOUS OUTCOME (EXAMPLE 1).
* HERE, THE OUTCOME IS SIMULATED AS BINARY (AS AN EXAMPLE).

*--------------------------------------------
* simpower-ex1-logit.do
    * Power simulation for Yij ~ [1+exp(-[mu + beta*Ai + bi])]^-1
            * (binary outcome with cluster (i) variability)
            * parameters:
            * tclust   : number of treatment clusters
            * cclust   : number of comparison clusters
            * nchild   : number of children per cluster
            * mu       : mean prevalence of the outcome in the control group
            * sdclust  : sd of random effect at the cluster level
            * or       : odds ratio (OR) of treatment:comparison
            * onesided : logical (one-sided test? default is two-sided)
*--------------------------------------------
capture program drop simpowerex1
program define simpowerex1, rclass
            version 9.0
            syntax [, tclust(real 100) cclust(real 100) nchild(real 20) mu(real 0) sdchild(real 0) sdclust(real 0.1) sdresid(real 1) or(real 1)
onesided]
* internal calculations
            local nclust   = `tclust'+`cclust'   /* total num clusters */
            local totobs   = `nchild'*`nclust'   /* total observations */
            local trobs    = `nchild'*`tclust'   /* total treatment observations */
            local obspercl = `nchild'            /* observations per cluster */
            local b0 = log(`mu'/(1-`mu'))        /* log-odds of the outcome in the comparison group */
            local b1 = log(`or')                 /* log of the odds ratio */
            if("`onesided'"=="onesided") local tail = 1
                        else local tail = 2
            local matsize  = `obspercl'+10
            set mat `matsize'
* create a child level dataset
            set obs `totobs'
            gen obsnum  = _n
            gen byte _x = 1 if mod(obsnum[_n-1],`obspercl')==0
            gen clustid = sum(_x)
* generate random effects for clusters
            sort clustid
            by   clustid: gen _rcl = invnormal(uniform())*`sdclust' if _n==1
            by   clustid: egen randclust = max(_rcl)
* assign treatment
            gen byte tr = (obsnum <= (`trobs'))
* simulate outcome
            gen double pr = (1+exp(-(`b0' + randclust + `b1'*tr)))^-1
            gen y = rbinomial(1,pr)
* run model and return results
            logistic y tr, cluster(clustid) robust
            return scalar beta = _b[tr]
            return scalar p    = `tail'*normal(-abs(_b[tr]/_se[tr]))
            drop _all
end simpowerex1

*--------------------------------------------
* Example : code check with null scenarios
* (should get ~5% power -- Type I error - due to two-sided p-value)
*--------------------------------------------
set seed 1029902
set more off
clear
#delimit;
simulate beta=r(beta) p=r(p), reps(10000): simpowerex1,
tclust(50) cclust(50) nchild(10) mu(0.1) sdclust(0.8) or(1);
#delimit cr
gen p05 = p<0.05
sum p05


*--------------------------------------------
* run simulations for cluster sizes 20(5)200,
* with 20 children per cluster
*--------------------------------------------

* open a postfile to store results
tempname memhold
tempfile results
postfile `memhold' clust child p using `results'

local nlist "20"
local clist "20(5)200"
foreach n of numlist `nlist' {
            foreach c of numlist `clist' {
                        clear
                        #delimit;
```

```
                        simulate beta=r(beta) p=r(p), reps(10000): simpowerex1,
                        tclust(`c') cclust(`c') nchild(`n') mu(0.1) sdclust(0.8) or(0.8);
                        #delimit cr
                        gen p05 = p<0.05
                        di as res _n "POWER FOR `n' CHILDREN PER CLUSTER, CLUSTER SIZE `c'"
                        sum p05
                        qui sum p05, meanonly
                        local p = r(mean)
                        post `memhold' (`c') (`n') (`p')
            }
}

postclose `memhold'
use `results', clear
outsheet using "~/dropbox/powersim/output/simpower-ex1-logit.csv", comma replace


exit
```

Example 3 (not presented in the text, an additional example...)
A parallel, longitudinal, cluster-randomized study with binary outcome

```
capture log close
set more off
clear mata
clear

* NOTE: THIS SIMULATION IS NOT PRESENTED IN THE TEXT,
* BUT PROVIDES AN EXAMPLE OF SIMULATING A BINARY OUTCOME
* IN A PARALLEL, LONGITUDINAL, CLUSTER RANDOMIZED STUDY (1 TREATMENT)

*--------------------------------------------
* simpower-ex3.do
* Power simulation for Yijt ~ [1+ exp(-[mu + beta*Ait + bi + bij])]^-1
* simulation for a binary outcome with cluster (i) and child (ij) level random effects
* allows for multiple visits (baseline + follow-up)
* parameters   :
            * tclust   : number of treatment clusters
            * cclust   : number of comparison clusters
            * nchild   : number of children per cluster
            * bvisit   : number of baseline (pre-intervention) measurements
            * fvisit   : number of follow-up (post-intervention) measurements
            * mu       : underlying mean probability of the outcome in the comparison group
            * sdchild  : sd of random effect at the child level
            * sdclust  : sd of random effect at the cluster level
            * or       : odds ratio (OR) of treatment:control
            * dropout  : proportion of post-baseline observations lost to follow-up
            * onesided : logical (one-sided test? default is two-sided)
            * (the returned value p is the 1- or 2-sided p-value for the test that OR=1)
*--------------------------------------------

capture program drop simpowerex3
program define simpowerex3, rclass
            version 9.0
            syntax [, tclust(real 100) cclust(real 100) nchild(real 20) bvisit(real 1) fvisit(real 1) mu(real 0.1) sdchild(real 0.1) sdclust(real 0.1)  or(real 1)
dropout(real 0) onesided ]
* internal calculations
            local nvisit   = `bvisit'+`fvisit'           /* total num visits */
            local nclust   = `tclust'+`cclust'           /* total num clusters */
            local totobs   = `nvisit'*`nchild'*`nclust'   /* total observations */
            local trobs    = `nvisit'*`nchild'*`tclust'   /* total treatment observations */
            local obspercl = `nvisit'*`nchild'           /* observations per cluster */
            local b0 = log(`mu'/(1-`mu'))                 /* log-odds of the outcome in the comparison group */
            local b1 = log(`or')                         /* log of the odds ratio (OR)*/
            if("`onesided'"=="onesided") local tail = 1
                        else local tail = 2
            local matsize  = `obspercl'+10
            set mat `matsize'
* create a child-visit level dataset
            set obs `totobs'
            gen obsnum = _n
            gen byte _x = 1 if mod(obsnum[_n-1],`obspercl')==0
            gen byte _y = 1 if mod(obsnum[_n-1],`nvisit')==0
            gen clustid = sum(_x)
            gen childid = sum(_y)
            bysort clustid childid: gen visit = _n
* generate random effects for clusters & children
            sort clustid
            by clustid: gen _rcl = invnormal(uniform())*`sdclust' if _n==1
            by clustid: egen randclust = max(_rcl)
            sort childid
            by childid: gen _rch = invnormal(uniform())*`sdchild' if _n==1
            by childid: egen randchild = max(_rch)
* assign treatment
            gen byte tr = (obsnum <= (`trobs')) & (visit > `bvisit')
* simulate a binary outcome
            gen double pr = (1+exp(-(`b0' + randclust + randchild + `b1'*tr)))^-1
            gen y = rbinomial(1,pr)
* account for dropout
            gen double u = uniform()
            drop if (u <= `dropout') & (visit > `bvisit')
* run model and return results
            logistic y tr, cluster(clustid) robust
            return scalar beta = _b[tr]
            return scalar p = `tail'*normal(-abs(_b[tr]/_se[tr]))
            drop _all
end simpowerex3



*--------------------------------------------
* Example : code check with null scenarios
* (should get ~5% power -- Type I error - due to two-sided p-value)
*--------------------------------------------
set seed 4100247
clear
#delimit;
simulate beta=r(beta) p=r(p), reps(10000): simpowerex3,
tclust(100) cclust(100) nchild(10) bvisit(1) fvisit(1) sdclust(0.8) sdchild(0.75) mu(0.1) or(1) dropout(0);
#delimit cr
gen p05 = p<0.05
sum p05
```

A technical note for programmers:

The Stata simulation code presented here can be sped up by around 30% by not using Stata's `simulate` command. Using the `simulate` command requires the simulation to create the design matrix in every iteration, which is inefficient. Instead, the simulations can be written by first creating the design matrix, and then looping over the random effect and outcome generation, using `postfile` to store results. We have provided the examples using the `simulate` command because we expect that the code is a bit more intuitive for less experienced programmers. Refer to the R code examples for implementations that create the design matrix once per simulation scenario.
